# A Push-Pull CORF Model of a Simple Cell with Antiphase Inhibition Improves SNR and Contour Detection

**DOI:** 10.1371/journal.pone.0098424

**Published:** 2014-07-24

**Authors:** George Azzopardi, Antonio Rodríguez-Sánchez, Justus Piater, Nicolai Petkov

**Affiliations:** 1 Johann Bernoulli Institute for Mathematics and Computer Science, University of Groningen, The Netherlands; 2 Institute of Computer Science, University of Innsbruck, Austria; CSIC-Univ Miguel Hernandez, Spain

## Abstract

We propose a computational model of a simple cell with push-pull inhibition, a property that is observed in many real simple cells. It is based on an existing model called Combination of Receptive Fields or CORF for brevity. A CORF model uses as afferent inputs the responses of model LGN cells with appropriately aligned center-surround receptive fields, and combines their output with a weighted geometric mean. The output of the proposed model simple cell with push-pull inhibition, which we call push-pull CORF, is computed as the response of a CORF model cell that is selective for a stimulus with preferred orientation and *preferred* contrast minus a fraction of the response of a CORF model cell that responds to the same stimulus but of *opposite* contrast. We demonstrate that the proposed push-pull CORF model improves signal-to-noise ratio (SNR) and achieves further properties that are observed in real simple cells, namely separability of spatial frequency and orientation as well as contrast-dependent changes in spatial frequency tuning. We also demonstrate the effectiveness of the proposed push-pull CORF model in contour detection, which is believed to be the primary biological role of simple cells. We use the RuG (40 images) and Berkeley (500 images) benchmark data sets of images with natural scenes and show that the proposed model outperforms, with very high statistical significance, the basic CORF model without inhibition, Gabor-based models with isotropic surround inhibition, and the Canny edge detector. The push-pull CORF model that we propose is a contribution to a better understanding of how visual information is processed in the brain as it provides the ability to reproduce a wider range of properties exhibited by real simple cells. As a result of push-pull inhibition a CORF model exhibits an improved SNR, which is the reason for a more effective contour detection.

## Introduction

Visual information is of great importance for humans and animals. In macaques, for instance, 55% of the neocortex is dedicated to process visual information [1], this is 5 to 20 times more than the resources dedicated to any other sensory information.

The study of [2]–[4] was the first breakthrough in the understanding of neurons in area V1 of the visual cortex. They distinguished three types of neurons that they called simple, complex and hypercomplex cells. Their work inspired many researchers to study and unveil the properties of other kinds of neurons in the same and other areas of the visual cortex [5], [6].

The visual cortex of the brain may be understood as being organized in a hierarchy [7], which is composed of layers of neurons that perform similar as well as varied operations. Neurophysiologists have identified two main pathways that process visual information, the so-called dorsal and ventral streams or as they are referred to, the “where” and “what” pathways, respectively. The dorsal stream is responsible for motion analysis and spatial arrangement while the ventral stream performs, essentially, object detection and recognition. The complexity of neuronal selectivity increases when going up the hierarchy. For instance, in the bottom layer of the ventral stream, neurons in area V1 respond to bars and edges, as well as spatial frequency, color, motion and disparity while at the higher end, neurons in area IT respond to whole objects independently of changes in location on the retina, stimulus size, contrast, color and aspect ratio (related to deph rotation invariance) [8], [9].

The ongoing findings of such neurophysiological studies have been the inspiration to computationally simulate how visual information is analyzed in the brain. During the last three decades, this has been the focus of many research groups in the computer vision community. Their work may not only contribute to more robust techniques but also to achieve a better understanding of how the brain processes visual information. Computational neuroscience and modeling address the big questions in computer vision by mimicking the human visual system as well as providing a ground where to test hypotheses on how the visual cortex works. In [10] the first approach was proposed to model some properties of simple and complex cells of the type reported by Hubel and Wiesel. Computational neuroscientists have been adding layers of functionalities to that pioneering work. Some of those works consist of modelling simple cells [11], as well as modelling hierarchies of simple and complex cells [12]. Other works have been adding new neural types and functionalities, such as the addition of lateral connections for contour grouping [13], [14] or neurons that are selective to shapes [15] to name a few among the extensive modeling bibliography.

In this work we focus on neurons in area V1 that respond to edges and bars. These neurons integrate responses of cells that reside in the lateral geniculate nucleus (LGN), an intermediate area between the eye and the visual cortex. In area V1, there are three main types of neuron that respond to bars and edges, referred to as simple, complex and hypercomplex neurons. A simple neuron responds to a bar or an edge of a given orientation at a specific position in its receptive field. A complex neuron is also orientation-selective but its response is invariant to the location of the preferred stimulus within its receptive field. It is usually considered as integrating responses from simple neurons [3] or LGN neurons [16]. Finally, hypercomplex (also known as end-stopped) cells are sensitive to the terminations of edges or bars [17].

The class of simple cells is the most studied type of neuron in neurophysiology, their detailed properties are very well known today. Besides orientation selectivity, they respond to gratings [5] and exhibit an orientation bandwidth which is invariant to the contrast of a stimulus. Another property that is typical of simple cells is called cross orientation suppression. This means that if two stimuli are presented at the same time, one of preferred orientation and the other one of orthogonal orientation, the response of the concerned simple cell decreases with increasing contrast of the orthogonally oriented stimulus [18].

While the 2D Gabor function [11] has gained particular popularity as a model of a simple cell, it fails to reproduce contrast invariant orientation tuning and cross orientation suppression. A novel computational model of a simple cell was proposed in [19], called CORF (Combination of Receptive Fields), that exhibits these two important properties. The authors demonstrated that the CORF model outperforms the Gabor function model in a contour detection task [20]. The response of that CORF model is based on excitatory synapses by a collection of afferent model LGN cells, the receptive fields of which are co-linearly aligned.

A CORF model takes as input the responses of a group of model LGN cells with center-surround receptive fields that are aligned along a row. The colinear arrangement of center-on receptive fields on one side and in parallel to a similar colinear arrangement of center-off receptive fields on the other side determines the orientation selectivity of a CORF model simple cell. This is in line with a recent exhaustive study [21], which found that the geometrical arrangement in the visual space of population receptive fields of geniculate inputs can predict the dominant orientation and spatial phase preferences of the simple cells in a cortical column. The response of a CORF model simple cell is computed as the weighted geometric mean of afferent LGN input. This AND-type operation follows the hypotheses of Hubel and Wiesel [22] as well as Marr and Hildreth [23] in that a simple cell fires only when all the afferent LGN cells with appropriately aligned receptive fields are activated. While the biological underlying mechanism is still an open research question, the AND-type operation proposed in the CORF model turned out to be essential to achieve contrast invariant orientation tuning and cross orientation suppression, as they could not be reproduced by an OR-type operation.

A classical receptive field of a simple cell is a region of the visual field where the presence of a visual stimulus with preferred contrast, size and orientation triggers the firing of the concerned cell. For instance, a simple cell that is selective for a vertical edge has a receptive field which is divided into two main areas, vertically oriented and elongated, parallel to each other, called the ON and OFF sub-regions. It fires when a vertical edge is within its receptive field and the light and dark parts of the stimulus are appropriately located on the ON and OFF sub-regions of the receptive field, respectively.

In neurophysiology, it is well known that simple cells receive what is called antiphase or push-pull inhibition [24]–[29]. A push-pull response of a simple cell with classical receptive field is achieved when two stimuli of preferred orientation but of opposite contrast evoke responses of the opposite sign; the stimulus of preferred contrast evokes a push (positive) response and the stimulus of opposite contrast evokes a pull (negative) response. Some simple cells are also known to have non-classical receptive fields [30]–[33] which receive inhibition from their surrounding. In [34] a computational model of a simple cell with surround inhibition was proposed, which is based on Gabor functions.

**Figure 1:**
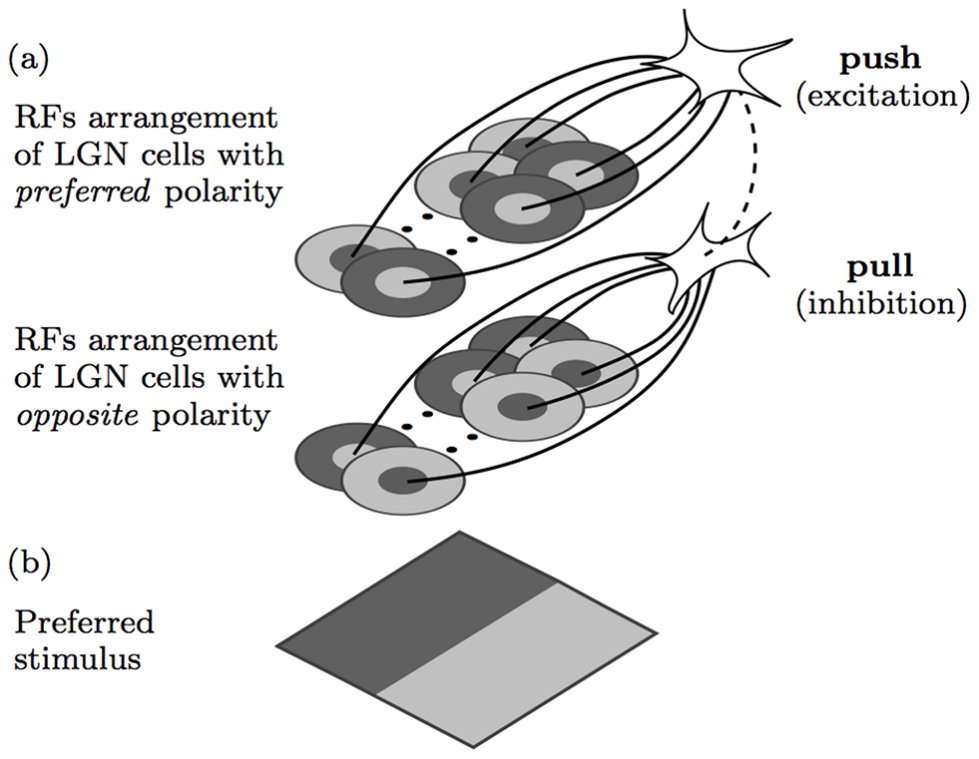
Model of push-pull inhibition. (a) Model of a (top) simple cell that receives excitatory or *push* input from model LGN cells with appropriately aligned receptive fields, and an inhibitory or *pull* input from another (bottom) cortical neuron that receives input from LGN cells with receptive fields of opposite polarity. Shaded light and dark gray areas indicate ON and OFF subregions, respectively, within the receptive fields of afferent model LGN cells. The solid lines indicate excitatory synaptic connections and the dashed line indicates an inhibitory synaptic connection. (b) Preferred stimulus that evokes maximum response to the concerned model.

A popular model of the push-pull response of a simple cell is depicted in Fig. 1. While there is not yet explicit biological evidence of the involved wiring it continues to receive strong neurophysiological experimental support [24], [26], [34]–[40]. It consists of a cortical neuron which receives excitation from a relay of thalamic LGN cells with center-surround receptive fields of preferred polarity, as well as inhibition from another cortical neuron, which receives input from LGN cells with center-surround receptive fields of opposite polarities.

There is neurophysiological evidence that push-pull inhibition is the most dominant form of inhibition received by simple cells [26], [29], [41]–[43]. This type of inhibition can be so strong that it may completely suppress the activation of a simple cell [41]. While the speculative feedforward push-pull model mentioned above has been evaluated with experimental data in neurophysiology, to the best of our knowledge, it has not yet been implemented as a computational model and evaluated in contour detection, which is assumed to be the biological role of simple cells.

We propose a push-pull CORF model of a simple cell with anitphase inhibition that takes as input the responses of two CORF model cells of the type proposed in [19], one with preferred polarity and the other one with opposite polarity, and compute its response as a function of the difference between their responses. We explore whether a push-pull CORF model exhibits the following two biological properties: separability of spatial frequency and orientation, and sensitivity of spatial frequency tuning to contrast [44], [45]. Moreover, we study the effectiveness of push-pull inhibition with regards to signal-to-noise ratio and to a contour detection application. We also compare this model with other biologically and non-biologically inspired contour operators.

The paper is organized as follows. First, we present the push-pull CORF model followed by experiments that demonstrate that it exhibits important properties of simple cells. Then, we present the experimental results in contour detection for two benchmark data sets of images with natural scenes. Finally, we provide a discussion about some aspects of the proposed model and draw our conclusions.

## Computational Model

### Overview

Fig. 1 illustrates the main setup of the push-pull CORF model of a simple cell that we propose. The concentric circles illustrate center-on (light central region with a dark surround) and center-off (dark central region with light background) receptive fields of model LGN cells. We use the CORF model that was proposed in [19] to model the colinear spatial arrangement of the receptive fields of model LGN cells. Its response is computed as the weighted geometric mean of the responses of the involved model LGN cells. The upper group of center-surround receptive fields is aligned in a colinear manner and with a polarity that is appropriate for the preferred stimulus shown at the bottom. The lower group corresponds to another CORF model which takes input from a group of model LGN cells of opposite polarity. Its response suppresses (or pulls) the excitatory (or push) response that is achieved with a CORF model of preferred polarity. The combined responses of these two model cells are then used to activate the corresponding model simple cell.

In the following sub-sections we explain the implementation details of the proposed push-pull CORF model.

### Implementation

**Figure 2:**
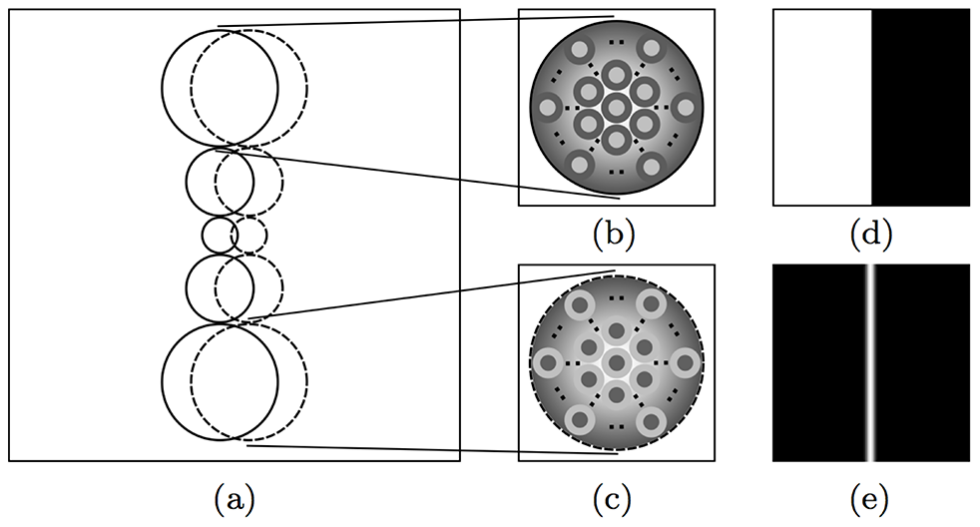
Receptive field and orientation selectivity. (a) The receptive field structure of a CORF model cell (of size 30×30 pixels). The solid and dashed circles represent sub-units that take as input the responses of center-on and center-off LGN model cells, respectively. (b) and (c) show a closer look at two types of sub-units. The image in (b) illustrates a sub-unit whose output is a Gaussian-weighted summation of the responses of a pool of center-on DoG functions, while the image in (c) illustrates a sub-unit that integrates center-off DoG responses. The radius of each sub-unit is a function that grows linearly with the Euclidean distance from the receptive field's center of the CORF model cell. (d) A synthetic stimulus (of size 100×100 pixels) of bright-to-dark vertical edge and (e) the corresponding response image obtained by sliding the CORF receptive field in (a) across all locations of the stimulus in (d).

We denote by *S* a CORF model simple cell that is selective for vertical edges, of the type shown in Fig. 2d, that we configure with the trainable method proposed in [19].
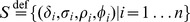
(1)where every four-tuple 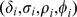 represents the properties of a pool of afferent model LGN cells, which we call sub-unit. We model an LGN cell by a difference-of-Gaussians (DoG) function, which has been evaluated many times in neuroscience as an appropriate model LGN cell [46]. In particular, *δ_i_* represents the polarity of the center-surround receptive fields (−1 for center-off, and 1 for center-on) of a pool of DoG functions, *σ_i_* represents the standard deviation of the outer Gaussian function of the involved DoG functions (the standard deviation of the inner Gaussian function is half of that of the outer Gaussian function), and 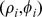 are the polar coordinates of the sub-unit's center with respect to the receptive field's center of the concerned CORF model cell.

The response of a CORF model cell at location 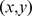, which we denote by 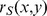, is achieved by combining the responses of the *n* afferent sub-units by weighted geometric mean. This computation is explained in detail in [19]. Fig. 2a illustrates the receptive field structure of a CORF model cell and Fig. 2e shows the response image that it achieves to the preferred stimulus shown in Fig. 2d.

The excitatory and inhibitory regions within the receptive field of a simple cell may either overlap or be separated in the direction orthogonal to the orientation preference of the cell [47]. We refer to the orthogonal distance between a pool of center-on and a pool of center-off model LGN cells as the separation index, which we denote by *B*. We consider the receptive field structure that results from the automatic configuration of a CORF model cell, such as the one shown in Fig. 2a, to have a separation index 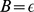. Below we study the properties of the model for values of the separation index larger than 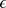: 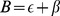.

**Figure 3:**
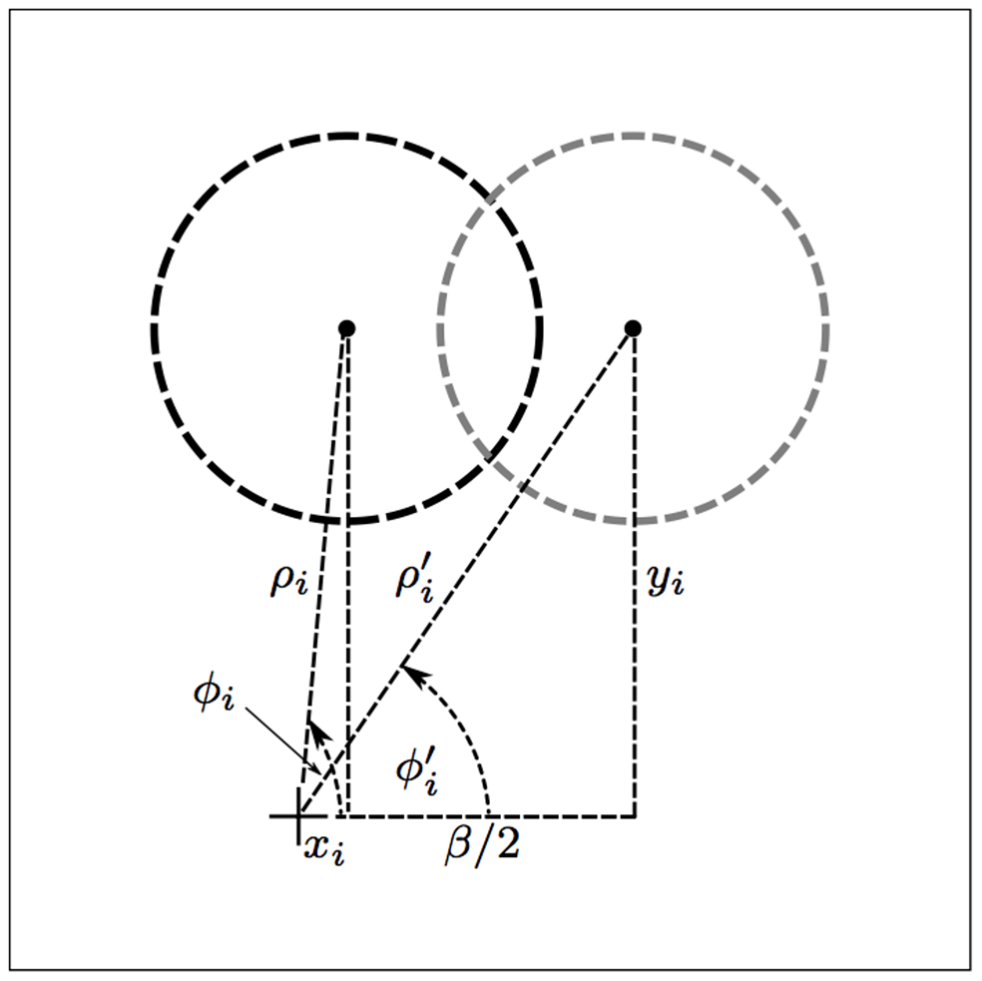
Automatic adjustment of a CORF receptive field for a given *β* value. The black and gray dashed circles represent the original and the shifted receptive field, respectively, of a center-off sub-unit that is described by tuple *i* in the concerned CORF model. The new polar coordinates (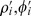), with respect to the ‘+’ marker (receptive field center of the CORF model at hand), are determined by shifting the polar coordinates (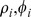) along the *x*-axis by half of the given *β* value.

From the set *S* that corresponds to 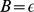, we form a new set 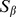 that defines another CORF model simple cell, which has the same preference for vertical orientations but has a separation index 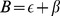:
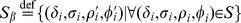
(2)where 
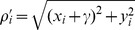
, 
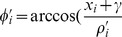
), 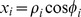, 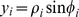, 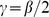 when 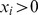 and 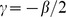 when 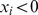. Fig. 3 illustrates the geometrical relationship between one pair of 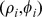 and its counterpart 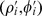.

**Figure 4:**
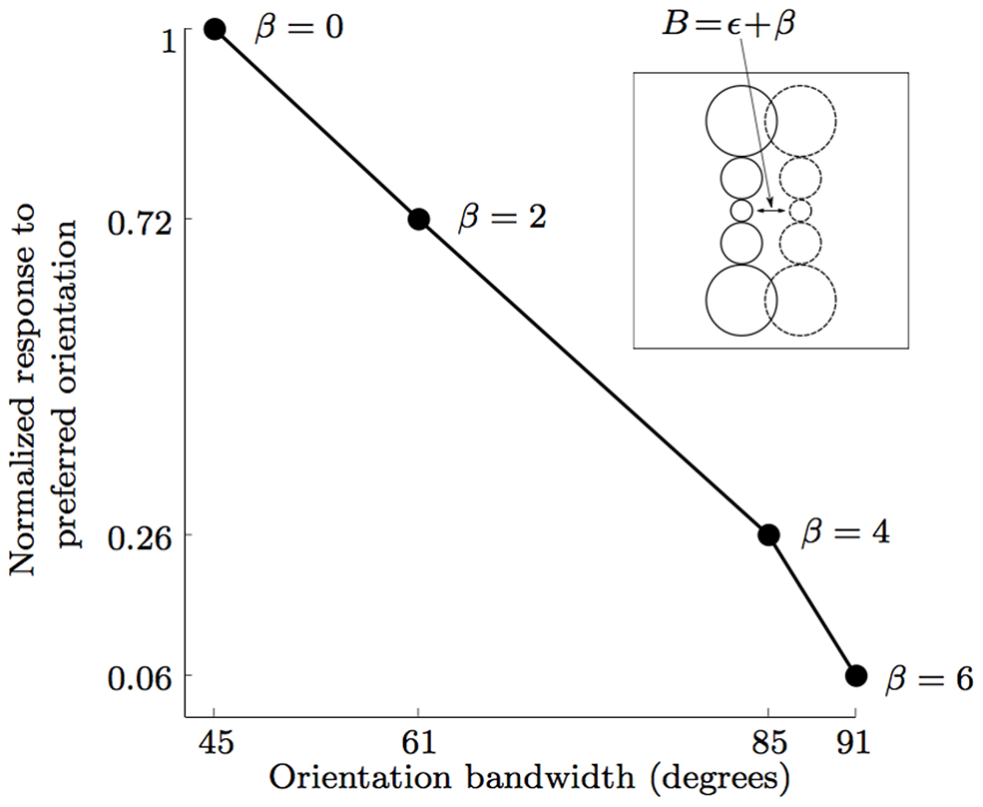
Relationship between the separation index *B* of the ON and OFF subregions of the receptive field of a CORF model cell (*see* inset) and the response to the preferred oriented edge and the orientation bandwidth at half amplitude. For *β* = 0 the ON and OFF sub-regions are organized as depicted in Fig. 2a. In this case, the concerned CORF model cell achieves maximum response with an orientation bandwidth at half amplitude of 45°. The orientation bandwidth increases and the response decreases with an increasing *β* value. The value of *β* for which the response disappears depends on the size of the pool - if it does not touch the edge, there will be no response.

The value of the parameter *β* effects the strength of the response to the preferred stimulus as well as the spatial frequency and orientation bandwidth of the concerned CORF model cell; the response to the preferred stimulus and the spatial frequency decreases, while the orientation bandwidth increases with an increasing value of *β*, Fig. 4.

We use set 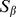 to define a new CORF model cell 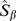 that is selective for vertical edges with opposite contrast:
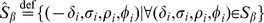
(3)


The receptive field of a CORF model 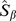 is in antiphase to the one of 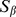. Push-pull inhibition is the result of combining the responses of two models, *S* (push) and 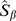 (pull), defined above. We use a non-negative *β* value only for the inhibitory part in order to achieve an orientation bandwidth that is broader than that of the excitation, a property that is supported by neurophysiological evidence [48], [49].

We denote by 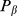 a push-pull CORF model simple cell and define it as a pair:
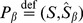
(4)


For *β*>0 the inhibitory CORF model has a smaller spatial frequency than the excitatory counterpart. An alternative way to achieve a similar effect is to use an inhibitory CORF model that has afferent model LGN cells with larger receptive fields (i.e. larger *σ* values) than those of the excitatory CORF model. We choose to work with the parameter *β* because it provides more flexibility to the model.

We compute the response of a push-pull CORF model cell at location (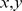) by subtracting a factor of the *pull* response 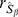 from the push response 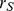, and denote it by 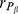:
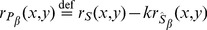
(5)where the parameter *k* represents the pull strength of the inhibition.

### Push-pull inhibition and signal-to-noise ratio

In the following we investigate the effect of push-pull inhibition on the signal-to-noise (SNR) ratio of computed neural responses. For this purpose we compare the SNR values of the responses of CORF models with and without inhibition to synthetic test images.

**Figure 5:**
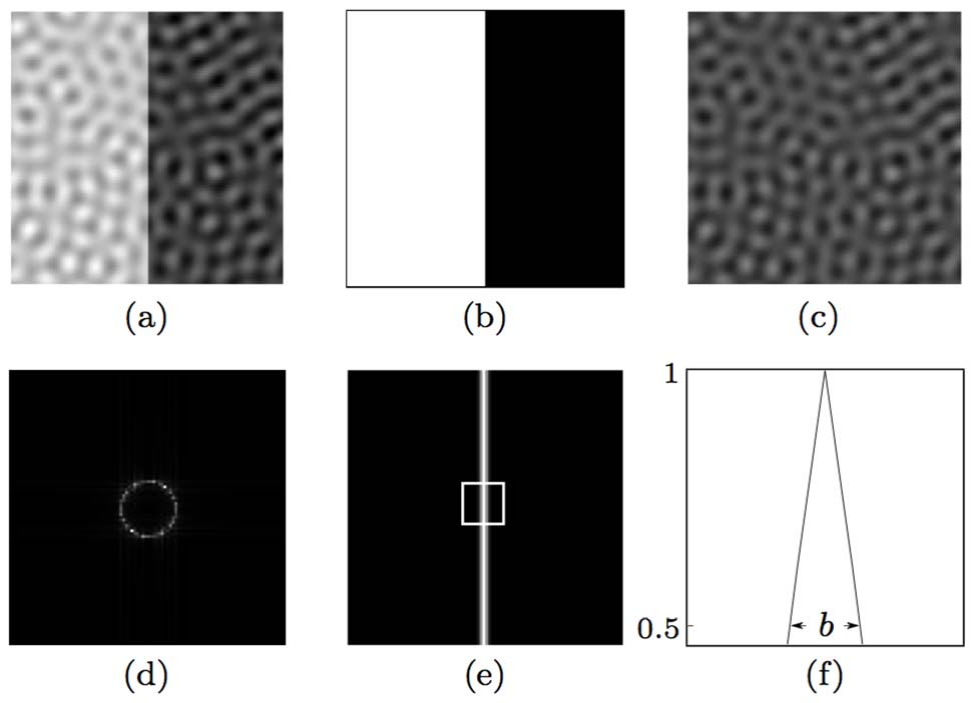
Construction of band-limited noisy images. (a) A test image (of size 100×100 pixels) is the sum of a (b) noiseless edge image and (c) a noise image. The noise image is a superposition of a constant value *N* (here *N* = 8) and 100 sinusoidal gratings of randomly selected orientations for the same spatial wavelength *w* (here *w* = 9 pixels). (d) The 2D spectrum of the noise image in (c). (e) Response map that is obtained by CORF model cells (with or without inhibition) to the preferred stimulus in (b). (f) A horizontal profile within the enframed region in (e). The label *b* (here *b* = 3 pixels) indicates the number of CORF responses at half amplitude along the horizontal direction, which is the direction orthogonal to the edge orientation.

We generate a test image by summing an image of a vertical bright-to-dark edge with full contrast and a noise image, Fig. 5(a–c). We use the method proposed by [50] to generate a band-limited noise image as a superposition of a constant value *N* and 100 sinusoidal gratings of randomly selected orientations, all with the same given spatial wavelength *w*. The rationale of using band-limited noise is that it is particularly effective for masking of contours due to the responses it elicits from orientation-selective model neurons. We set the amplitude of the gratings as one third of the given average noise luminance *N*. The resulting test image has an edge contrast *C* defined as *C* = 1/*N*.

Fig. 5e illustrates the response map obtained by a CORF model cell without inhibition to the preferred stimulus shown in Fig. 5b. For the same noiseless stimulus an equivalent result is achieved by a push-pull CORF model cell that we propose. The maximum responses are achieved along the edge and they rapidly decrease with an increasing deviation from the edge until they disappear. The label *b* in Fig. 5f indicates the width of the band around the edge that contains responses greater than half of the maximum response.

We create nine test images by using three contrast values (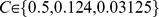) and three values of *w* (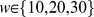). For all the locations of a test image we apply two CORF model cells, one without inhibition and the other with push-pull inhibition (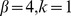) and obtain two response maps. For this experiment both CORF models have the common parameter *σ* set to 2 and they both result in a band of width 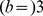 pixels to a noiseless edge stimulus of preferred orientation.

For each map, we then compute the average of the responses of a model cell along the band of width *b* that surrounds the edge and call it the response to signal 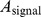. Similarly, we compute the average of the responses of the same model cell in the remaining noisy areas and call it the response to noise 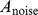. Finally, we compute the SNR in decibels as follows:
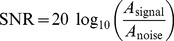
(6)


**Figure 6:**
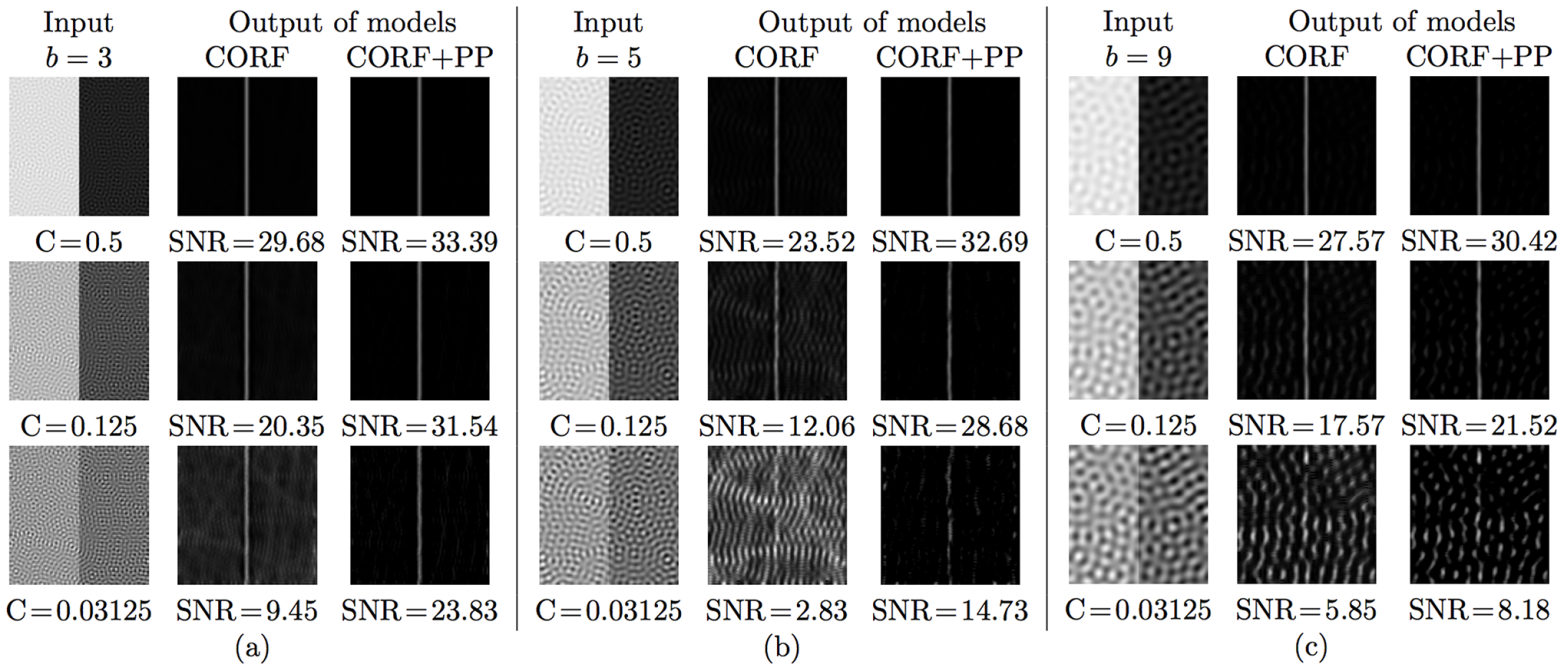
Experimental results of the SNR obtained with CORF model cells with no inhibition (CORF) and with push-pull inhibition (CORF+PP). The first columns of (a–c) contain test images that are obtained by varying the spatial wavelength *w* and the contrast value *C* of band-limited noise. The second and third columns of (a–c) are the response maps obtained by the concerned CORF and CORF+PP model cells, respectively. A CORF model cell with push-pull inhibition systematically exhibits an improved SNR.

Fig. 6 shows the synthetic test images that we use along the corresponding response maps that are obtained with the two types of CORF model cells. These experimental results clearly show that the proposed push-pull CORF model cell improves the SNR substantially.

### Tolerance to Rotation

The model configured above has an orientation preference for bright-to-dark vertical edges, Fig. 2(d–e). This preference is determined from a user-specified prototype edge by a configuration process that is thoroughly explained in [19]. We form a new set 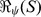 that describes a CORF model simple cell to be selective for edges that have an orientation of *ψ* radians:
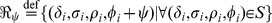
(7)


In order to obtain a response that is tolerant to any orientation we take the maximum value of push-pull CORF models with different orientation preference at a given location (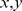):
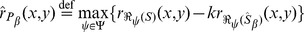
(8)where Ψ is a set of 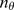 orientations: 
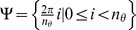
. A value of 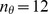 is sufficient as a push-pull CORF model cell achieves an orientation bandwidth at half amplitude of 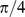, Fig. 4.

## Testing Some Properties of Simple Cells

### Separability of spatial frequency and orientation

The majority of simple cells exhibit an orientation tuning that is separable (or independent) of spatial frequency [51]. However, there are other cells whose orientation tuning is affected by the spatial frequency of a stimulus [44], [45].

**Figure 7:**
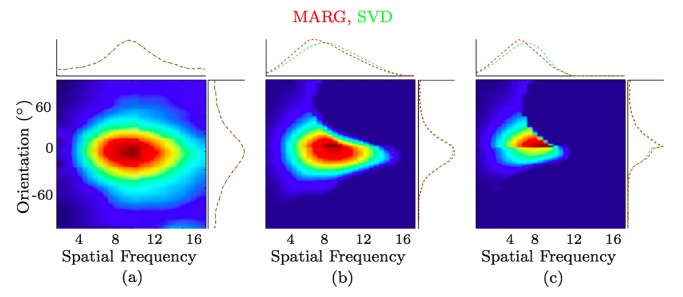
Separability of spatial frequency and orientation. (a) Response maps of a CORF model cell without inhibition (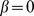, 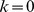), to gratings of different spatial frequency and orientation, (b) with moderate push-pull inhibition (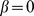, 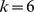) and (c) with strong inhibition (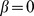, 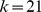) The red and green plots indicate the marginal (MARG) row- and column-wise sums, and singular value decomposition (SVD), respectively. These results are comparable to the response of biological cells (see Fig. 3 in [51]).

We explore the separability properties of the proposed push-pull CORF model. Fig. 7a shows a response map of a CORF model cell without inhibition (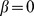, 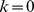) to gratings of different frequency and orientation. We computed two measurements, 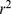 and *si*, that were used in [51] in order to quantify the separability between spatial frequency and orientation. The quantity 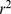 is the squared correlation between measured and predicted spatial frequency-orientation. Predicted values are obtained under the assumption that both features (spatial frequency and orientation) are independent. The other quantitity 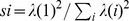 is related to how much the first singular vector reconstructs the original matrix after singular value decomposition. Both quantities range between 0 (non-separable) to 1 (separable). We refer to [51] for further technical details on the rationale of these quantities. We obtained a value of 0.96 for 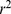 and a value of 0.99 for *si*. Such high values (very close to 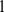) mean that the spatial frequency and orientation are almost perfectly separable. Fig. 7b shows a response map which we obtain by adding moderate inhibition (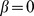, 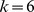), and it results in 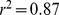 and 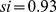. This scenario is very similar to the average over 52 neurons reported in [51]. Fig. 7c shows another response map for much stronger inhibition (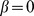, 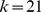), which results in 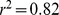 and 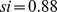. These experiments indicate that the separability of spatial frequency and orientation tuning decreases as the inhibition strength increases.

**Figure 8:**
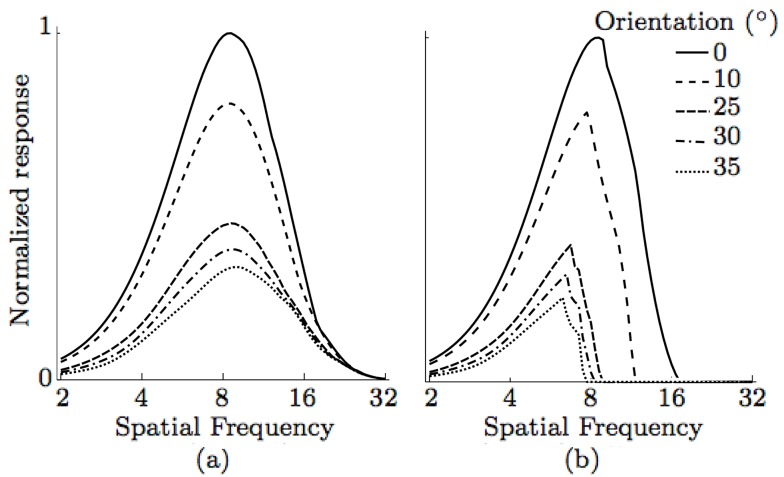
Relationship of spatial frequency and orientation selectivity. (a) A CORF model cell without inhibition (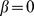, 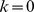) has independent relations between the preferred spatial frequency and orientation, while (b) a CORF model cell with push-pull inhibition (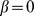, 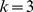) shows a dependent relationship. This is similar to what is observed in biological simple cells (see Fig. 1 in [45]).

The studies in [45] and [51] share a common finding; they report that some simple cells whose preferred spatial frequency varies with orientation and other cells whose preferred spatial frequency is independent of the orientation of the grating. Next, we demonstrate how we can achieve both phenomena with the proposed model by simply changing the push-pull inhibition factor *k* in Eq. 8. In Fig. 8 we show the activity of the proposed model that achieves comparable behaviour to the two most extreme cases from the work of [45]. When no inhibition is applied (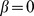, 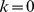) we obtain a model cell whose preferred spatial frequency is completely independent of the grating orientation (top) as in the case of simple cell 3 studied in [45]. On the other hand, if we add push-pull inhibition (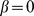, 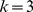) (bottom) we obtain a model cell whose preferred spatial frequency is dependent on the orientation of the grating as in cell 16 studied in [45].

### Spatial frequency tuning sensitive to contrast

Some simple cells in visual cortex have a spatial frequency tuning that is sensitive to contrast [52]. We can also achieve this property by incorporating a sublinear function, such as the sigmoid function, to the responses of model LGN cells that provide input to CORF model cells.

**Figure 9:**
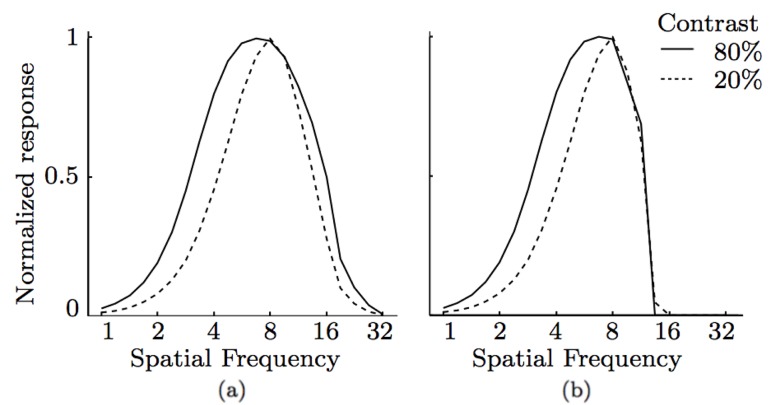
Spatial frequency sensitive to contrast. Spatial frequency tuning curves as a function of contrast obtained by two CORF model cells; (a) with no inhibition (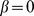, 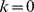) and (b) with push-pull inhibition (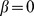, 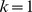). The dependence of spatial frequency tuning and contrast changes is achieved only when the model LGN cells are processed by a sigmoid function.

The resulting CORF model cells with and without inhibition show dependence of spatial frequency tuning to contrast, Fig. 9.

## Application to Contour Detection

In the following, we evaluate the proposed push-pull CORF model in a contour detection task. First, we explain how we transform a given image of a natural scene into a binary contour map and then we present a quantitative procedure to evaluate the quality of the resulting contour map.

Finally, we compare the performance of the proposed model to several other computational models, including the basic CORF model without inhibition, the Gabor Filter model of a simple cell with and without surround inhibition, the Gabor energy model of a complex cell with and without surround inhibition, as well as to the classical Canny edge detector.

### Data sets and ground truth

**Figure 10:**
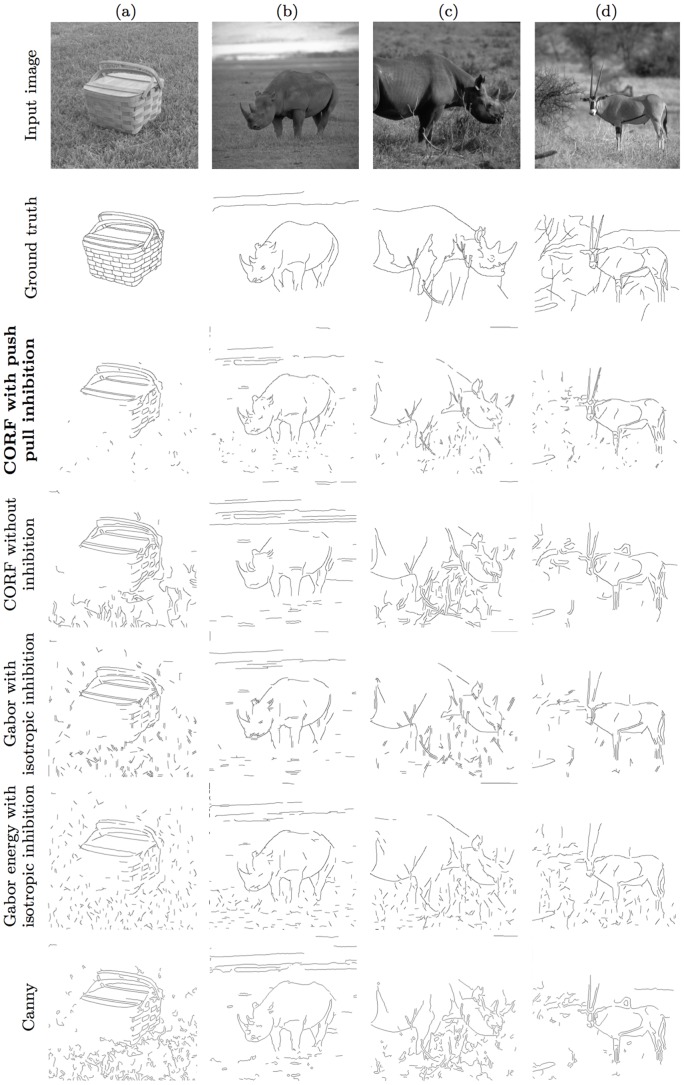
Examples of RuG images, their ground truth and the respective contour maps obtained by five operators. (First row) Images of objects in natural scenes taken from the RuG data set. (Second row) The corresponding contour maps hand drawn by a person. Best contour maps obtained by (third row) the proposed push-pull CORF model, (fourth row) the basic CORF model without inhibition, (fifth row) the Gabor filter model with isotropic surround inhibition, (sixth row) the Gabor energy model with isotropic surround inhibition and by (seventh row) the classical Canny edge detector.

We use two benchmark data sets that were created by the Universities of Groningen (RuG: the data set is online: http://www.cs.rug.nl/~imaging) and Berkeley. The RuG data set was originally introduced in [53] for the evaluation of the Gabor (energy) filter model with non-classical receptive field. It consists of 40 colour images (of size 512×512 pixels) of objects in natural scenes. Fig. 10 (first row) illustrates four examples of images taken from this data set, and Fig. 10 (second row) illustrates the corresponding ground truth contour maps that are hand drawn by a person. The ground truth images depict only the contours of objects (and shadows) and omit the sporadic contours of textured background.

**Figure 11:**
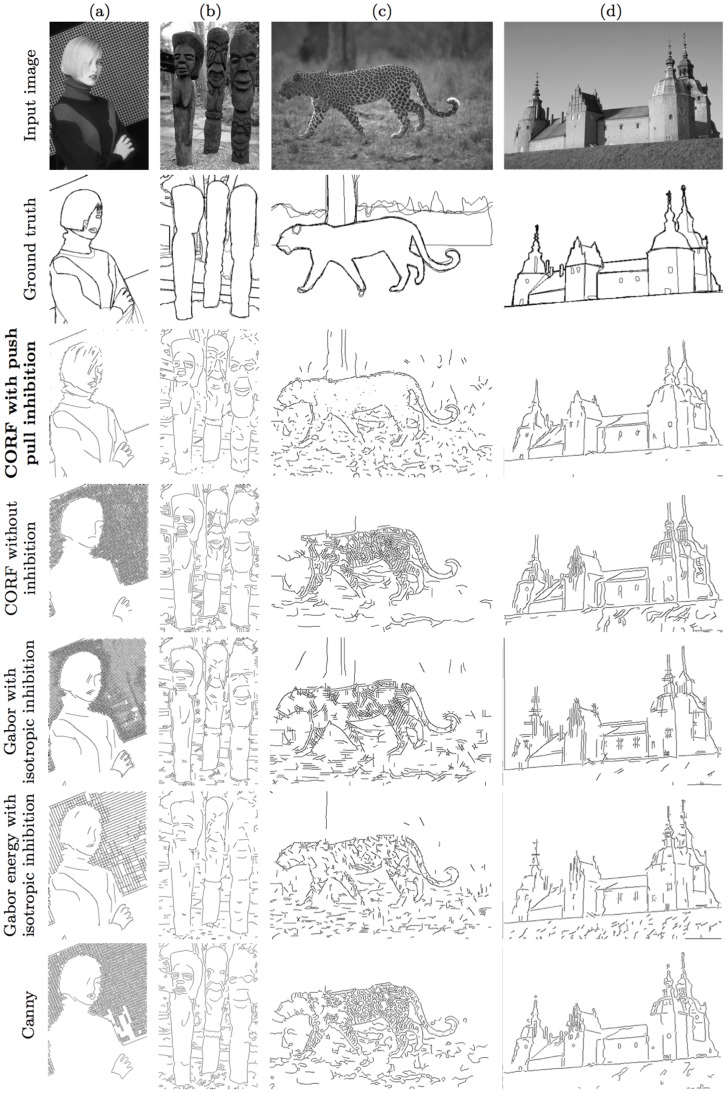
Examples of Berkeley images, their ground truth and the respective contour maps obtained by five operators. (First row) Images of objects in natural scenes taken from the Berkeley data set. (Second row) The corresponding collection of superimposed contour maps hand drawn by five persons. Best contour maps obtained by (third row) the proposed push-pull CORF model, (fourth row) the basic CORF model without inhibition, (fifth row) the Gabor filter model with isotropic surround inhibition, (sixth row) the Gabor energy model with isotropic surround inhibition and by (seventh row) the classical Canny edge detector.

The Berkeley data set consists of 500 images (of size 481×321 or 321×481 pixels) of objects in complex scenes. Fig. 11 (first row) shows four examples of images taken from this data set. While this data set was mainly developed for the evaluation of segmentation algorithms, it has also been used to evaluate various contour detection operators. Each image in the Berkeley data set is complemented with a collection of five ground truth contour maps which were hand drawn by five different persons. Fig. 11 (second row) illustrates the ground truth of superimposed contour maps that correspond to the images in the first row. The bolder the contour is the better the agreement is among the involved human observers.

Next, we explain how we obtain binary contour maps from the operators that we use here for comparison. Subsequently, we define the performance measures that we use to quantify the quality of the resulting contour maps with respect to the given ground truth images.

### Binary contour map

We apply a classical two-step procedure in computer vision that was proposed by [54] and [55] to obtain a binary contour map from the output of the concerned model. The first step consists of edge thinning by non-maximum suppression to determine the ridges in the given response image. Then, we apply hysteresis thresholding to obtain a binary contour map. The latter step requires a high and a low threshold value. Similar to the work in [19] we set the low threshold value to a fraction (0.5) of the high threshold. For a given image, we set the high threshold to be the lowest value of the strongest *ζ* pixels in the thinned response image. The given value of the parameter *ζ* is a fraction of the total number of pixels in the image. The resulting binary map contains the strongest fraction *ζ* of contour pixels together with any connected ones that are achieved by hysteresis thresholding.

The images in the third to the seventh row of Fig. 10 and of Fig. 11 show the contour maps of the proposed push-pull CORF model, the basic CORF model without inhibition, the Gabor and Gabor energy models with isotropic surround inhibition and the classical Canny edge detector for the RuG and Berkeley data sets, respectively. These maps are obtained for certain values of the high threshold parameter that are explained below.

### Quantitative performance measure

A binary contour map consists of two unbalanced sets of pixels, a minority set of contour pixels and a majority set of non-contour pixels.

We use the Matthews' correlation coefficient (*mcc*) as a quantitative measure to compare such unbalanced binary maps, which are obtained by some contour operators, with the corresponding ground truth. This performance measure, which is appropriate even when the concerned classes are unbalanced, considers the number of correctly detected contour pixels (true positives or *TP*), the number of pixels that are incorrectly detected as contour pixels (false positives or *FP*), the number of correctly detected background pixels (true negatives or *TN*) and the number of incorrectly missed contour pixels (false negatives or *FN*):
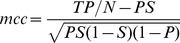
(9)where 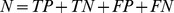, 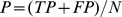, and 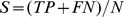.

The *mcc* values vary between −1 and +1. A value of +1 means perfect prediction, a value of 0 means random prediction, while a value of −1 indicates a completely wrong prediction.

We use the method described by [19] to deal with inexact contour localizations between the given ground truth and binary contour maps.

### Experimental setup

In our experiments we perform various evaluations and comparisons. First, we determine the best *β* value and inhibition factor *k* for the model that we propose. This is achieved by running a systematic set of experiments on the RuG data set, each time using a different combination of the following parameters: 21 values of the scale parameter (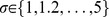), five *ζ* values (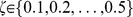), five *β* values (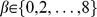 and 15 inhibition factors (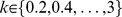). For 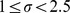 we use three radii (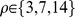), for 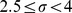 we use four radii (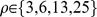) and for 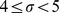 we use five radii (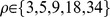). These *ρ* values are selected in such a way that the resulting orientation bandwidth at half amplitude is 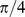. For each combination of 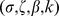 parameters we compute the mean *mcc* (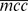) value for all the 40 images in the RuG data set. The maximum 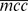 is achieved for 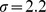, 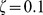, 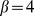 and 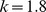. The contour maps shown in Fig. 10 (third row) are obtained with these parameter values. For the Berkeley data set we do not search for the best *β* and *k* parameter values but we use the same ones (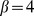, 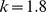) that were determined from the RuG data set.

Next, we compare the proposed push-pull CORF-based operator (CORF+PP) to the basic CORF-based operator without inhibition. This experiment allows us to understand the effectiveness of the addition of push-pull inhibition. Furthermore, we compare our model with an alternative inhibitory model of a simple cell called Gabor filter with isotropic surround inhibition (GF+II). For the sake of completeness, we also make a comparison with the Gabor energy filter model with isotropic inhibition (GEF+II), which is a computational model of a complex cell in area V1 with non-classical receptive field inhibition. For the Gabor-based operators [53] showed that isotropic surround inhibition is more effective in contour detection than anisotropic surround inhibition. Finally, we compare our results with the classical Canny edge detector.

The five operators that we compare share a common parameter, namely the scale parameter *σ*. For the CORF-based operators *σ* represents the standard deviation of the outer Gaussian function of the DoG filters that provide input, for the Gabor-based operators it represents the standard deviation of the envelope Gaussian function and for the Canny edge detector it represents the standard deviation of a Gaussian smoothing kernel.

For the Gabor-based operators (GF+II, GEF+II), we set the wavelength 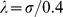 and the spatial aspect ratio 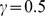 as suggested by [56]. Furthermore, we set the inhibition factor 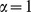 of the Gabor-based operators as it yielded the maximum 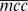 value for the RuG data set. We consider 12 orientations (in intervals of 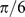) for the CORF- and Gabor-based operators.

## Results

For every input image we apply the above five mentioned contour detection operators with 21 different values of the parameter *σ* (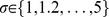) and five values of the parameter *ζ* (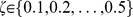).

Finally, we compute the 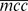 value for each value combination of parameters *σ* and *ζ* and for each data set. Table 1 reports the parameter values of *σ* and *ζ* that contribute to the maximum 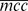 value. In the fourth to the seventh row of Fig. 10 and Fig. 11 we show the binary contour maps of the CORF, GF+II, GEF+II and Canny operators with the parameter values reported in Table 1 for the RuG and Berkeley data sets, respectively.

**Table 1:** The best parameters for the five evaluated operators.

	CORF+PP		CORF		GF+II		GEF+II		Canny	
	*σ*	*ζ*	*σ*	*ζ*	*σ*	*ζ*	*σ*	*ζ*	*σ*	*ζ*
**RuG**	2.2	0.1	4.8	0.1	3.6	0.1	2.8	0.2	2.4	0.1
**Berkeley**	2.2	0.3	3.6	0.2	3.4	0.3	2.0	0.3	2.0	0.2

The values of parameters *σ* and the fraction *ζ* of minimum pixels (from thinned images) to generate the resulting binary contour maps, which contribute to the maximum 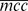 for the operators that we apply to the RuG and Berkeley data sets.

**Figure 12:**
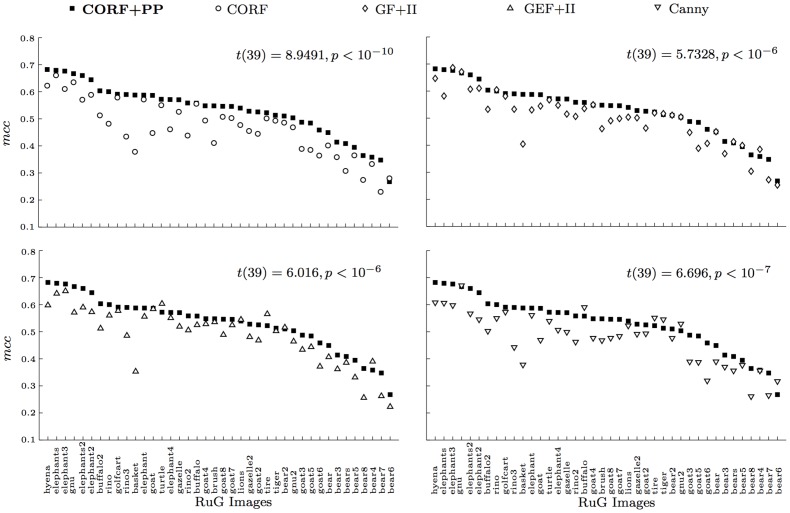
Comparison of contour detecton results to the images of the RuG data set. The proposed push-pull CORF model outperforms CORF (without inhibition), Gabor function with isotropic inhibition (GF+II), Gabor energy function with isotropic inhibition (GEF+II) and the Canny edge operators in the majority of the cases.

Fig. 12 shows four scatter plots that illustrate pairwise comparisons between the proposed CORF+PP operator with the other four state-of-the-art operators for the RuG data set. The labels in the *x*-axis are the RuG image names in descending order of the corresponding *mcc* value that is achieved with the proposed push-pull CORF model. We compare the *mcc* values of each image that are achieved with the values of parameters *σ* and 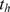 reported in Table 1. For the majority of the images, the proposed operator achieves a better *mcc* value. In particular, out of the 500 images of the Berkeley data set, the proposed CORF+PP operator achieves better performance in 434, 377, 451, and 437 cases in comparison to the CORF-based operator without inhibition, GF+II, GEF+II and Canny edge detector, respectively.

On a statistical level, we apply a right-tailed paired-samples *t*-test to the set of pairs of *mcc* values that are achieved by the proposed CORF+PP operator and by each of the other four operators. The CORF+PP operator that we propose outperforms all other operators with high statistical significance for both the RuG (CORF: 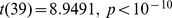, GF+II: 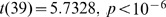, GEF+II: 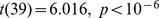, Canny: 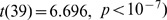 and the Berkeley (CORF: 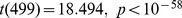, GF+II: 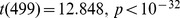, GEF+II: 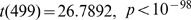, Canny: 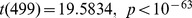) data sets.

In order to test the generalization ability of the above experimental method, we perform a 10-fold cross validation on the Berkeley data set. For each fold we consider nine different sets of 50 images and for each operator we apply a grid search to determine the *σ* and *ζ* parameter values that contribute to the maximum average *mcc* score across the (9×50 = ) 450 training images. It turns out that for all the 10 folds and for each operator we achieve the same *σ* and *ζ* parameter values as reported in Table 1 on the entire data set of 500 images. This result demonstrates the generalization ability of the applied experimental setup. Moreover, the fact that for the Berkeley data set we use the *β* and *k* parameter values that were determined from the RuG data set demonstrates the generalization ability of the proposed CORF detector with push-pull inhibition.

In an iterative procedure we perform a grid search to every possible combination of 9 sets of images, such that in each iteration we leave a different set out of consideration. This procedure is performed for the five operators. For the 10 grid searches, the threshold parameters of the operators remain constant and match the ones reported in Table 1 for the whole data set. The scale parameter remains constant only for the proposed CORF detector with push-pull inhibition (sigma = 2.2), GF+II (sigma = 3.4) and GEF+II (sigma = 2). For the basic CORF operator without inhibition the scale parameter is 3.6 for six grid searches and 3.8 for the remaining four. For the same six and four grid searches the scale parameter of the Canny operator is set to 2 and 2.2, respectively.

## Discussion

In contrast to other computational models of simple cells, in particular the ones that rely on the Gabor function [11] and difference-of-Gaussians [15], [57]–[59], the proposed push-pull CORF model cell is anatomically more realistic as it uses as afferent inputs the responses of model LGN cells, rather than intensity pixels as projected on the retina.

In other studies we demonstrated that by using orientation-selective filters as afferent inputs we can form models that achieve qualitatively similar responses to shape-selective neurons in area V4, and showed that such models can be effectively used in various computer vision applications [60], [61].

The push-pull CORF model cell that we propose differs from the Gabor-based models with non-classical receptive field inhibition (nCRF) in two main aspects. First, the proposed model uses *one* model cell with *opposite polarity* to provide inhibition to the concerned model simple cell. Second, the receptive fields of the inhibitory neuron and simple cell models *overlap* each other. For 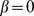 there is a complete overlap, and for 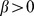 the receptive field of an inhibitory model neuron expands in all directions from the center, resulting in a bigger receptive field than that of the simple cell but with the same center. To the contrary, nCRF models receive inhibition as a function of the total responses of many model cells that are outside (no overlap) the receptive field of the model cell at hand. This is also known as contextual modulation.

In previous work [19], it was shown that a CORF model without inhibition exhibits contrast invariant orientation tuning, cross orientation suppression and response saturation, three properties that are typical of simple cells. Here, we demonstrate that by adding push-pull inhibition we can extend the number of properties that are observed in real simple cells. These include the relationship between spatial frequency and orientation tuning and spatial frequency selectivity that is sensitive to contrast. As a matter of fact, push-pull inhibition may be at the heart of an ongoing discussion in neurophysiology. A CORF model without inhibition exhibits orientation tuning that is *independent* of spatial frequency [51], but when we add push-pull inhibition the resulting model exhibits less separability between orientation tuning and spatial frequency. Similarly, by changing the strength of push-pull inhibition we can control the sensitivity of contrast to spatial frequency.

We demonstrated by quantitative experiments that the addition of push-pull inhibition improves signal-to-noise ratio systematically. This is the reason why a contour operator based on the proposed model outperforms the one without inihibition with high statistical significance. The highest improvement is achieved in images with high textured (noisy) background, such as the images shown in Fig. 10(a–c), Fig. 11a and Fig. 11c. For images that consist of only perceptually salient objects without noise, the result will be the same. The contour detection experiments also demonstrate that the proposed implementation of push-pull inhibition is more effective than Gabor-based models with nCRFs. Similarly, it outperforms the popular Canny edge detector.

The proposed model is conceptually simple and easy to implement. A push-pull response is computed as the response of a CORF model with preferred polarity minus a factor of the response of another CORF model with the same orientation but opposite polarity.

## Conclusions

Push-pull inhibition provides the ability to construct models of a wider range of real simple cells with various properties that cannot be reproduced by other computational models. Besides orientation selectivity, cross-orientation suppression, contrast-invariant orientation tuning and response saturation, the proposed method can be used to implement a model cell whose relationships between its selectivity for spatial frequency, orientation tuning and contrast can be controlled by the strength of push-pull inhibition.

In addition, a push-pull CORF model cell improves SNR substantially, and outperforms other brain-inspired (Gabor-based) contour operators and the classical Canny edge detector.
